# Mouse Genome Database (MGD): Knowledgebase for mouse–human comparative biology

**DOI:** 10.1093/nar/gkaa1083

**Published:** 2020-11-24

**Authors:** Judith A Blake, Richard Baldarelli, James A Kadin, Joel E Richardson, Cynthia L Smith, Carol J Bult, the Mouse Genome Database Group, Anna V Anagnostopoulos, Jon S Beal, Susan M Bello, Olin Blodgett, Nancy E Butler, Jeffry Campbell, Karen R Christie, Lori E Corbani, Mary E Dolan, Harold J Drabkin, Maria Flores, Susan L Giannatto, Angelina Guerra, Paul Hale, David P Hill, Jonathan Judd, Meiyee Law, Monica McAndrews, David Miers, Cailey Mitchell, Howie Motenko, Li Ni, Hiroaki Onda, Janice Ormsby, Michelle Perry, Jill M Recla, David Shaw, Dmitry Sitnikov, Monika Tomczuk, Lauren Wilming, Yunxia ‘Sophia’ Zhu

**Affiliations:** The Jackson Laboratory, Bar Harbor, ME, USA; The Jackson Laboratory, Bar Harbor, ME, USA; The Jackson Laboratory, Bar Harbor, ME, USA; The Jackson Laboratory, Bar Harbor, ME, USA; The Jackson Laboratory, Bar Harbor, ME, USA; The Jackson Laboratory, Bar Harbor, ME, USA; The Jackson Laboratory, Bar Harbor, ME, USA

## Abstract

The Mouse Genome Database (MGD; http://www.informatics.jax.org) is the community model organism knowledgebase for the laboratory mouse, a widely used animal model for comparative studies of the genetic and genomic basis for human health and disease. MGD is the authoritative source for biological reference data related to mouse genes, gene functions, phenotypes and mouse models of human disease. MGD is the primary source for official gene, allele, and mouse strain nomenclature based on the guidelines set by the International Committee on Standardized Nomenclature for Mice. MGD’s biocuration scientists curate information from the biomedical literature and from large and small datasets contributed directly by investigators. In this report we describe significant enhancements to the content and interfaces at MGD, including (i) improvements in the Multi Genome Viewer for exploring the genomes of multiple mouse strains, (ii) inclusion of many more mouse strains and new mouse strain pages with extended query options and (iii) integration of extensive data about mouse strain variants. We also describe improvements to the efficiency of literature curation processes and the implementation of an information portal focused on mouse models and genes for the study of COVID-19.

## INTRODUCTION

As the cost of genome-scale sequencing continues to decrease and new technologies for genome editing become widely adopted, the laboratory mouse is more important than ever as a model system for understanding the biological significance of human genetic variation and for advancing the emergence of genomic medicine. The Mouse Genome Database (MGD) (1) has a unique and strategic role as a community resource for facilitating the use of the laboratory mouse for understanding the genomics underlying human biology and disease. MGD serves three major user communities: (i) biomedical researchers who use mouse experimentation to investigate genetic and molecular principles of biology and disease processes, (ii) translational scientists who use the laboratory mouse to model human disease and (iii) bioinformaticians/computational biologists who use the rich integrated data MGD provides to develop algorithms and bioinformatics tools for data analysis and interpretation. MGD processes and data support and comply with FAIR principles (2) with a variety of formats for data distribution.

MGD maintains a comprehensive catalog of mouse genes and genome features connected to genomic sequence data and biological annotations. As the community model organism knowledgebase for the laboratory mouse, MGD contains comprehensive information about mouse gene function, genotype-to-phenotype annotations, and mouse models of human disease. Annotations include: (i) molecular function, biological process and cellular location of gene products using terms and relations from the Gene Ontology (GO) (3), (ii) mouse mutations, variants and human disease models with genotypes annotated to the Mammalian Phenotype Ontology (MP) (4) and the Disease Ontology (DO) (5) and (iii) standardized nomenclature and identifiers for mouse gene names, symbols, alleles and strains. The rigorous application of nomenclature and annotation standards in MGD ensures that the information in the resource is curated consistently to support robust and comprehensive data retrieval for accurate identification of genes that share biological properties and data mining for knowledge discovery (Table 1).

**Table 1.** Data for which MGD serves as the authoritative source

Data type	Community relationship
Unified genome feature catalog	MGD compares/integrates predictions from Ensembl, NCBI, Havana/Vega, produces unified catalog used by NCBI, IMPC.
Gene Ontology (GO) annotations for mouse	MGD does primary curation, integrates data from others, provides definitive mouse GO annotation sets to GO site.
Mouse Phenotype annotations	MGD does primary curation & integrates data from publications & large scale projects.
Mouse models of human diseases	MGD does primary mouse model curation using disease terms & human gene associations from OMIM and NCBI.
Gene-to-nucleotide sequence association	Co-curation with MGA (Mouse Genome Annotation) group.
Gene-to-protein sequence association	Co-curation with UniProt and Protein Ontology.
Mammalian Phenotype (MP) Ontology	MGD develops & distributes MP. MP is actively used by many groups, e.g., RGD, MRC Harwell, Sanger, IMPC, etc.
Symbols & names for genes & genome features	MGD provides access to International Nomenclature guides, implements policies, coordinates with human and rat groups.
Strain designations	MGD assigns official nomenclature; provides to repositories.

MGD is a core resource within the Mouse Genome Informatics (MGI) consortium (http://www.informatics.jax.org). Other MGI consortium databases include the Gene Expression Database (GXD) (6), the Mouse Models of Human Cancer Database (MMHC; formerly the Mouse Tumor Biology database) (7), the Gene Ontology project (3), MouseMine (8), the International Mouse Strain Resource (IMSR) (9) and the CrePortal database of recombinase expressing mice (http://www.informatics.jax.org/home/recombinase). Data included in all resources hosted at the MGI website are obtained through a combination of expert curation of the biomedical literature and automated or semi-automated processing of data sets downloaded from more than fifty other data resources. A summary of the current content of MGD is presented in Table 2.

**Table 2.** Summary of MGD content September 2018–2020 [data as of 8 September 2020]

Data type	2018	2020
Number of genes and genome features with nucleotide sequence data	49 244	50 053
Number of genes with protein sequence data	24 408	24 278
Number of mouse genes with human orthologs	17 094	17 098
Number of mouse genes with rat orthologs	18 512	18 506
Number of genes with GO annotations	24 581	24 610
Total number of GO annotations	316 240	431 755
Number of mutant alleles in mice	56 254	64 571
Genes with mutant alleles in mice	13 455	14 999
Number of QTL	6605	7402
Number of genotypes with phenotype annotation (MP)	62 551	68 394
Total number of MP annotations	326 292	351 064
Number of mouse models (genotypes) associated with human diseases	6374	6912
Number of references in the MGD bibliography	258 926	287 019

In this report we describe significant enhancements to MGD, including two new graphical user interfaces: (i) improvements to the Multiple Genome Viewer for exploring the genomes of multiple mouse strains (ii) enhancement and inclusion of information of now over 64 000 strains of mice including collaborative cross strains and (iii) incorporation of SNP data with extensive updates in query and visualization of variant × strain data. Other improvements include improvements to literature curation processes, and specific access to data about mouse models for the study of coronavirus infections.

## NEW FEATURES AND CURATION WORKFLOW ENHANCEMENTS

### Enhancements to the multiple sequence viewer

**Figure 1.**
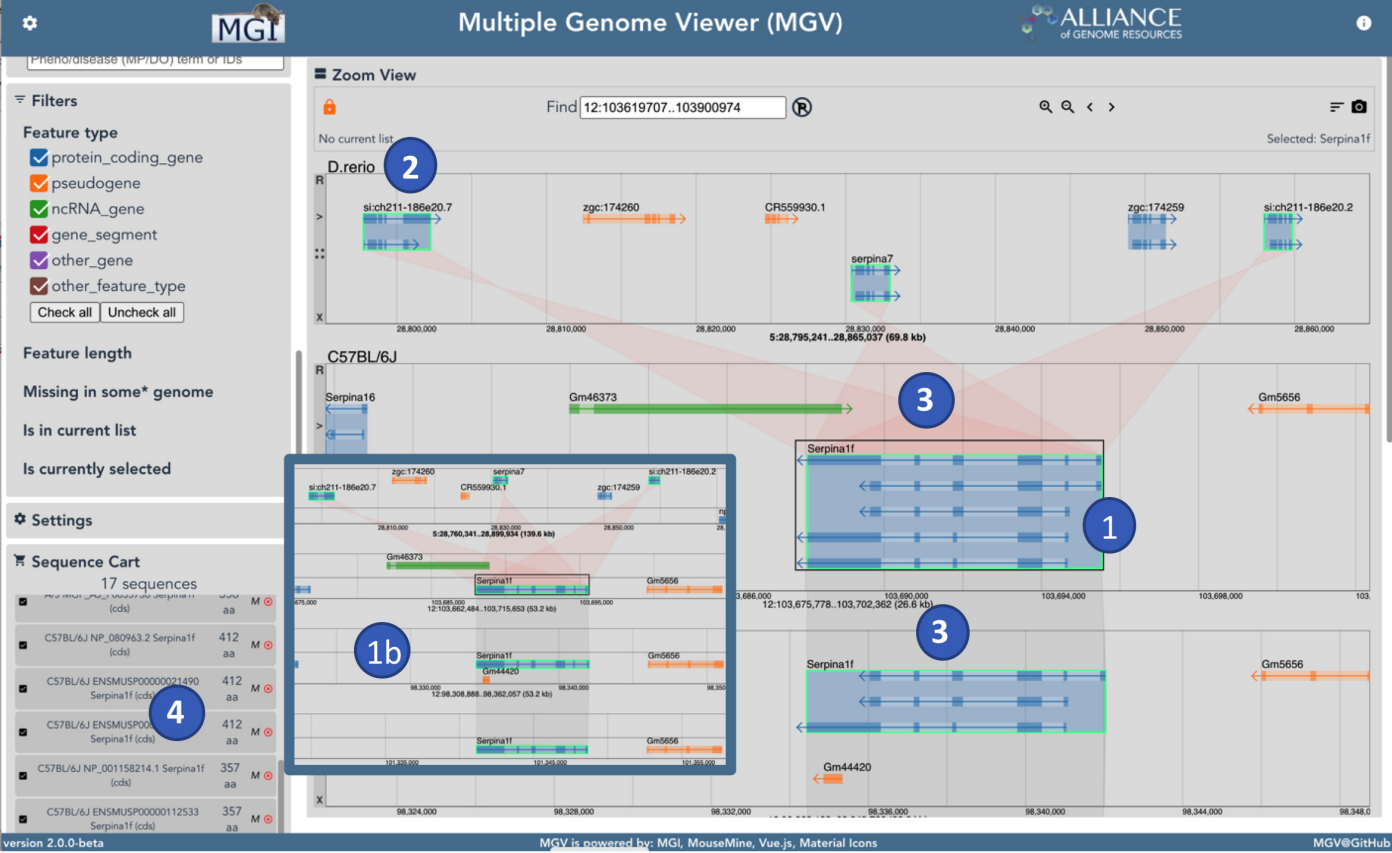
This screenshot shows several recent enhancements to MGV. (1) Intron/exon structure is shown for all transcripts of a gene, although the user can also switch to a compact view (1b) showing only a representative transcript for each gene; (2) organisms from the Alliance of Genome resources have been added, including human, rat, zebrafish (shown), fly, worm and yeast; (3) the Alliance strict orthology data set is used for connecting genes across organisms, while common canonical gene id is used to connect genes across strains and (4) the user can select and download sequences in FASTA format. Here the user has selected ‘all CDS sequences for Serpin1f and its displayed orthologs

The Multiple Genome Viewer (MGV, http://www.informatics.jax.org/mgv) is a web-based browser designed for navigating and comparing multiple related genomes simultaneously. The first release of MGV in 2018 included nineteen sequenced and annotated inbred strains of mice. It allowed the user to select the strains to view, designate one as the ‘reference’, and then support coordinated navigation by mapping navigation coordinates in the reference genome to coordinates in all the others. We have recently released version 2, which includes major enhancements to MGV’s data content and comparative navigation capabilities (Figure 1) as follows:

- Displays complete gene models (all transcripts and their intron/exon structure) and allows zooming down to the sequence level.
- Supports selection and downloading of sequences from the currently displayed genomes and/or from specific genes or transcripts.
- Goes beyond mouse. Now includes annotated genomes from the Alliance of Genome Resources for human, rat, fish, fly, worm and yeast.
- Uses orthology data from the Alliance (the strict set) for asserting homology across genomes. This is combined with use of ‘canonical’ identifiers to assert homology between genes in strains within a species. The interface directly supports viewing and navigating complex orthology relationships.
- Uses orthology relationships to infer paralogs, which are used to return additional genes in certain contexts, and to draw additional connections between displayed genes. Users can turn this feature on/off.
- The user has many controls to adjust the display, such as specifying which gene labels are displayed, whether to display all transcripts or just one representative per gene, the ability to download the current display as an PNG or SVG file, and more.

### New mouse strain data pages

**Figure 2.**
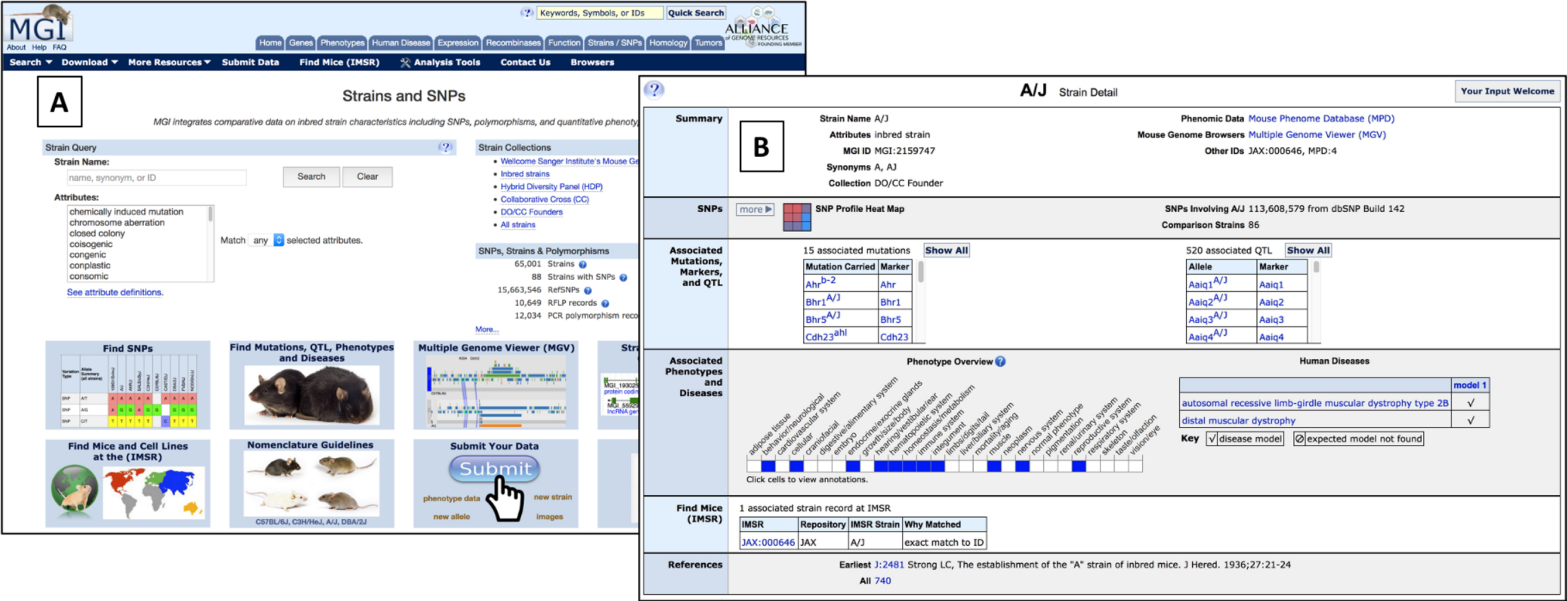
(**A**) Strain and SNPs home page and search form and (**B**) strain detail page for the A/J inbred mouse strain.

New mouse strain data pages are now available, including information for over 64 000 inbred, mutant congenic and co-isogenic strains, Collaborative Cross strains, recombinant inbred and other mouse strain types (Figure 2). The newly revamped Strain and SNPs mini-home page (http://www.informatics.jax.org/home/strain) includes a search form for mouse strains by nomenclature or by strain attribute, quick links to lists of strain collections such as the Collaborative Cross, and links to tools and strain resources.

Strain detail pages show standardized nomenclature, unique identifiers, repository stock IDs and synonyms. In addition, the new pages have a SNP profile heat map and links to detailed SNP data for 88 inbred strains, links to associated mutation records carried by the strain at MGI, and QTL associated with inbred strains. Links are also available to data at the Mouse Phenome Database (MPD) (10) for baseline quantitative strain measurements and the Multiple Genome Viewer (MGV) for comparing multiple strain and species genomes. Expertly curated disease model and qualitative strain phenotype characteristics are summarized at a glance in ribbon display format with more details available by clicking boxes in the ribbon. In addition to data from literature sources and the conversion of the former MGD text pages ‘Characteristics of inbred strains of Mice and Rats’ by M. Festing (11), we have already incorporated strain data from multiple mouse mutant repositories including JAX, MMRRC, EMMA, RIKEN and many others via the International Mouse Strain Resource (IMSR, http://www.findmice.org) into MGD. IMSR is a searchable online catalog of mouse strains, stocks, and mutant ES cell lines available worldwide. Links are provided at the bottom of the MGD strain detail pages to repository information and availability status at the IMSR. We will continue to improve integration of supporting strain data in MGD, loading these strain data and continuing to expertly curate new strains and data from the literature. As well, we will continue collaborative work with the Mouse Phenome Database (MPD, https://phenome.jax.org) to create better integration between our resources.

### Strain group comparisons for murine SNP data

**Figure 3.**
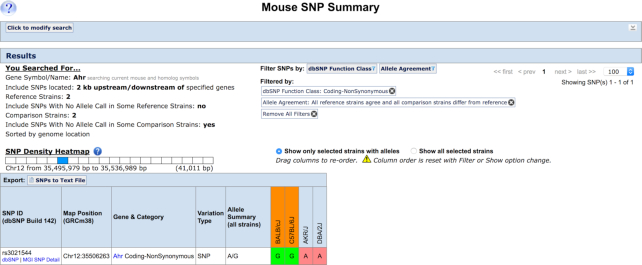
Strain group comparisons of SNPs. Screenshot of the SNP search results page showing the only non-synonymous coding SNP in the *Ahr* gene were the variant in BALB/cJ and C57BL/6J (strains with high ligand binding affinity) differs from AKR/J and DBA/2J (strains with low ligand binding affinity). This SNP (rs3021544) has been shown to cause a reduction in ligand binding.

MGD contains almost 16 million SNPs from 88 strains of mice. To support the use of SNP variation data to identify causative genes and variants, we implemented functionality that allows comparisons of SNPs for groups of strains with phenotypic differences. For example, inbred strains differ in their response to treatment with non-halogenated polycyclic aromatic hydrocarbons via the aryl-hydrocarbon receptor gene (*Ahr*). AKR/J and DBA/2J inbred strains carry non-responsive alleles (lower ligand binding affinity) while BALB/cJ and C57BL/6J harbor responsive alleles (4-fold higher affinity). Figure 3 shows the results of SNP search and filter options for the *Ahr* gene where the variant results in a non- synonymous coding change in the *Ahr* protein and where the SNP alleles are the same in AKR/J and DBA/2J but different than BALB/cJ and C57BL/6J. Of 114 SNPs associated with the *Ahr* gene involving these four strains, only a single SNP matches the search criteria. This SNP (rs3021544) has been shown to cause a reduction in ligand binding and is the causative variant (12,13). Following the decision of NCBI’s dbSNP to no longer support variation data from model organisms, MGD will begin sourcing mouse SNP data from the European Variation Archive (EVA; https://www.ebi.ac.uk/eva/).

### Enhanced efficiencies for literature triage and curation

The subset of papers about mice that are relevant to MGD (i.e. those focused on genetics and genomics of the laboratory mouse) continues to remain relatively stable year by year. In each of 2018 and 2019 we curated ∼12 000 papers primarily from a core set of 120 journals. Our literature acquisition process involves using full text search for the word ‘mice’ from this core set of journals to find potentially relevant articles which are then downloaded as PDF files. Automated processes extract text from the PDFs, populate bibliographic metadata from PubMed for the references, and keyword searches on the extracted text support curation procedures.

Recent process improvements include automating full text search and download from PubMed Central for nine relevant open access journals and automating a heuristic algorithm for splitting the extracted text into sections (body, references, manuscript figures, STAR methods, supplemental data). This allows the reference section to be omitted from keyword searches (e.g. if a paper only contains ‘mice’ in the predicted reference section, it is considered not relevant for curation).

### Contributions to the alliance of genome resources

MGD is one of the founding members of the Alliance of Genome Resources, a new data resource integration effort among the major model organism (MOD) database groups and the Gene Ontology Consortium (GOC) (14,15). MGD contributes core data to the Alliance including, specifically, mouse genome and phenotype data, expression data, and variant data. MGD’s most recent contributions to the Alliance have focused on the contributions to new representations and inclusion of disease model data, requirements analysis for Alliance-wide management of the biomedical literature, and development of new data structures and alignments to support access to variant data in a comparative context. MGD genetic and genomic data for the laboratory mouse are available from the public web portal for the Alliance of Genome resources (http://www.alliancegenome.org) and the Alliance links back to primary data in MGD in multiple instances.

### Development of mouse models for COVID-19 resource

The emergence and spread of the novel zoonotic coronavirus, SARS-CoV-2, in 2019 has led to global scientific efforts to understand the biology of this virus and to develop effective treatments and vaccines to combat the human disease, COVID-19. To streamline access to expertly curated studies of coronavirus infection, pathology and treatment in mice, MGD implemented the Mouse Genome Informatics Coronavirus Information portal (http://www.informatics.jax.org/mgihome/other/coronavirus.shtml). The portal aggregates information about murine research resources for SARS-CoV-2 and other coronaviruses. Data accessible from the portal includes curated publications from 1980-present, lists of mouse models and their availability in repositories around the world, and human and mouse genes relevant to coronavirus research.

## IMPLEMENTATION AND PUBLIC ACCESS

The production database for MGD is a highly normalized relational database hosted on a PostgreSQL server behind a firewall. The production database is designed and optimized for data integration and incremental updating and is not accessible by the public. The public web interface is backed by a combination of highly unnormalized databases (also in PostgreSQL) and Solr/Lucene indexes, designed for high performance query and display in a read-only environment. The front-end data stores are refreshed weekly from the production database. The separation of public and production architectures provides a flexibility in project planning, as either side can change without affecting the other. The MGD system runs on a large collection of virtual servers in the local cloud hosted at The Jackson Laboratory.

MGD broadcasts data in a variety of ways to support basic research communities, clinical researchers and advanced users interested in programmatic or bulk access. MGD provides free public web access to data from http://www.informatics.jax.org. The web interface provides a simple ‘Quick Search’, available from all web pages in the system and is the most used entry point for users. The Quick Search may be used to search for genes and genome features, alleles, and ontology or vocabulary terms. Multi-parameter query forms for a number of data types are provided to support searches based on specific user-driven constraints, Genes and Markers; Phenotypes, Alleles and Diseases; SNPs; and References. Data may be retrieved from most results pages by downloading text or Excel files, or forwarding results to Batch Query or MouseMine analysis tools (see below).

MGD offers batch querying interfaces for data retrieval for users wishing to retrieve data in bulk. The Batch Query tool (http://www.informatics.jax.org/batch) is used for retrieving bulk data about lists of genome features. Feature identifiers can be typed in or uploaded from a file. Gene IDs from MGI, NCBI GENE, Ensembl, VEGA, UniProt and other resources can be used. Users can choose the information set they wish to retrieve, such as genome location, GO annotations, list of mutant alleles, MP annotations, RefSNP IDs and Disease Ontology (DO) terms. Results are returned as a web display, in tab delimited text or Excel format. All data is freely available. Results may also be forwarded to MouseMine (see below).

MGD data access is available through MouseMine (http://www.mousemine.org) (8), an instance of InterMine that offers flexible querying, templates, iterative querying of results and linking to other model organism InterMine instances. MouseMine access is also available via a RESTful API, with client libraries in Perl, Python, Ruby, Java and JavaScript. MouseMine contains many data sets from MGD, including genes and genome features, alleles, strains and annotations to GO, MP and DO, and the complete annotated genomes for 19 inbred mouse strains.

MGD provides a large set of regularly updated database reports from http://www.informatics.jax.org/downloads/. Direct SQL access to a read-only copy of the database is also offered (contact MGI user support for an account). MGI User Support is also available to assist users in generating customized reports on request. Finally, the entire database is available for download as a PostgreSQL dump file.

Interactive graphical interfaces for browsing mouse genome annotations is supported through our instance of JBrowse (http://jbrowse.informatics.jax.org/?data=data/mouse), a JavaScript-based interactive genome browser with multiple features for navigation and track selection (16). As well, the Multiple Genome Viewer (MGV http://informatics.jax.org/mgv) provides for simultaneous, homology-driven exploration of 19 inbred strains of mice and other genomes represented in the Alliance.

## FUTURE DIRECTIONS

In addition to continuing the essential core functions of MGD, three major enhancements are planned for upcoming development.

First, we will update all genome feature coordinates in MGD with the most recent assembly of the C57BL/6J reference genome (GRCm39). This is the first coordinate-changing revision to the mouse reference assembly in over 8 years by The Genome Reference Consortium (17), and, compared to the previous assembly, delivers notable improvements in contiguity, gap count and average gap length, (GRCm38). In particular, the Build 39 assembly features a significant retiling of the pseudoautosomal region (PAR), and now provides distinct coordinates for the PARX and the PARY regions. MGD will reconcile our unified genome feature catalog (18) to Build 39, as updated genome annotation files from our providers become available. This process will result in creation of new genes and genome features in MGD, as well as removal of obsoleted features that were predicted from annotations of Build 38. Lastly, MGD will stay current with updates to the genome assemblies of mouse inbred strains and their annotations as these are released from Ensembl and Gencode, replacing the source files with the updated versions for the MGI.gff3 file, MGV and MouseMine and then updating the representation of mouse strain genes from the updated MGI.gff3 file. The next set of updated files are expected to be available by the end of this year. These updated assemblies and their annotations then are incorporated into the Multiple Genome Viewer (MGV) for convenient access.

Second, we will extend our representation of phenotypic variants and comparative views of human-mouse phenotype and disease model data. We are currently migrating structured text descriptions of mutant mouse alleles to genome sequence-based notations using Human Genome Variation Society (HGVS) standards and plan to update allele and genome data displays and provide programmatic data access to this information. These data are already available at the Alliance of Genome Resources. In order to expand disease model representation, we will also incorporate the disease model association predictions from human data alongside our experimentally validated annotations on our gene and genotype-phenotype annotation detail pages as well as in the Human-Mouse Disease Connection.

Third, we will refresh our representation of orthology, and consequent comparative views of human and mouse genetics and genomics. We will incorporate the ‘strict’ orthology generated by the algorithms implemented in the Alliance of Genome Resources project. We will provide both mouse to human and human to mouse ortholog and paralog relationship working with the Alliance team to align and standardize these data. We will continue to provide access to other comparative orthology resources such as HCOP (19), giving users information about additional vertebrate genomes.

Overall, we will continue to integrate genetic, genomic, and biological data critical for using the mouse as an experimental model for human biology and disease by maintaining and enhancing MGD as a resource for computational biologists, for translational, clinical and for bench scientists.

## OUTREACH

User Support staff are available for on-site help and training on the use of MGD and other MGI data resources. MGD provides off-site workshop/tutorial programs (roadshows) that include lectures, demos and hands-on tutorials and can be customized to the research interests of the audience. To inquire about hosting an MGD roadshow, email mgi-help@jax.org. On-line training materials for MGD and other MGI data resources are available as FAQs and on-demand help documents.

Members of the User Support team can be contacted via email, web requests, phone or fax.

- World wide web: http://www.informatics.jax.org/mgihome/support/mgi_inbox.shtml
- Facebook: https://www.facebook.com/mgi.informatics
- Twitter: https://twitter.com/mgi mouse and https://twitter.com/hmdc_mgi
- Email access: mgi-help@jax.org
- Telephone access: +1 207 288 6445

MGI-LIST (http://www.informatics.jax.org/mgihome/lists/lists.shtml) is a forum for topics in mouse genetics and MGI news updates. It is a moderated and active email-based bulletin board for the scientific community supported by the MGD User Support group. MGI-LIST has over 1200 subscribers. A second list service, MGI-TECHNICAL-LIST, is a forum for technical information about accessing MGI data for software developers and bioinformaticians, for using the APIs and for making web links to MGI pages.

## CITING MGD

For a general citation of the MGI resource, researchers should cite this article. In addition, the following citation format is suggested when referring to datasets specific to the MGD component of MGI: Mouse Genome Database (MGD), MGI, The Jackson Laboratory, Bar Harbor, Maine (URL: http://www.informatics.jax.org). Type in date (month, year) when you retrieved the data cited.

## MOUSE GENOME DATABASE GROUP

Anna V. Anagnostopoulos, Jon S. Beal, Susan M. Bello, Olin Blodgett, Nancy E. Butler, Jeffry Campbell, Karen R. Christie, Lori E. Corbani, Mary E. Dolan, Harold J. Drabkin, Maria Flores, Susan L. Giannatto, Angelina Guerra, Paul Hale, David P. Hill, Jonathan Judd, Meiyee Law, Monica McAndrews, David Miers, Cailey Mitchell, Howie Motenko, Li Ni, Hiroaki Onda, Janice Ormsby, Michelle Perry, Jill M. Recla, David Shaw, Dmitry Sitnikov, Monika Tomczuk, Lauren Wilming and Yunxia ‘Sophia’ Zhu.
